# Investigating antimalarial drug interactions of emetine dihydrochloride hydrate using CalcuSyn-based interactivity calculations

**DOI:** 10.1371/journal.pone.0173303

**Published:** 2017-03-03

**Authors:** Holly Matthews, Jon Deakin, May Rajab, Maryam Idris-Usman, Niroshini J. Nirmalan

**Affiliations:** Environment and Life sciences, University of Salford, Greater Manchester, United Kingdom; Stanford University, UNITED STATES

## Abstract

The widespread introduction of artemisinin-based combination therapy has contributed to recent reductions in malaria mortality. Combination therapies have a range of advantages, including synergism, toxicity reduction, and delaying the onset of resistance acquisition. Unfortunately, antimalarial combination therapy is limited by the depleting repertoire of effective drugs with distinct target pathways. To fast-track antimalarial drug discovery, we have previously employed drug-repositioning to identify the anti-amoebic drug, emetine dihydrochloride hydrate, as a potential candidate for repositioned use against malaria. Despite its 1000-fold increase in *in vitro* antimalarial potency (ED_50_ 47 nM) compared with its anti-amoebic potency (ED_50_ 26–32 uM), practical use of the compound has been limited by dose-dependent toxicity (emesis and cardiotoxicity). Identification of a synergistic partner drug would present an opportunity for dose-reduction, thus increasing the therapeutic window. The lack of reliable and standardised methodology to enable the *in vitro* definition of synergistic potential for antimalarials is a major drawback. Here we use isobologram and combination-index data generated by CalcuSyn software analyses (Biosoft v2.1) to define drug interactivity in an objective, automated manner. The method, based on the median effect principle proposed by Chou and Talalay, was initially validated for antimalarial application using the known synergistic combination (atovaquone-proguanil). The combination was used to further understand the relationship between SYBR Green viability and cytocidal versus cytostatic effects of drugs at higher levels of inhibition. We report here the use of the optimised Chou Talalay method to define synergistic antimalarial drug interactivity between emetine dihydrochloride hydrate and atovaquone. The novel findings present a potential route to harness the nanomolar antimalarial efficacy of this affordable natural product.

## Introduction

The prioritisation of combinatorial regimes over monotherapy in malaria has meant that the analysis of drug interactions has become an increasingly important part of the drug development pipeline [1, 2]. Due to the limited number of chemotherapies available, not least because the development of two novel compounds is doubly challenging, candidates are usually combined with existing treatments [3, 4]. This strategy delays resistance acquisition and thereby prolongs the shelf-life of the valuable cohort of current drugs. The ideal partner drugs for malaria treatment should have matching pharmacokinetics, synergise against drug-resistant strains, be free of significant side-effects, be relatively inexpensive and have no other antimicrobial activity [4, 5]. The values of combination therapy, namely: (i) better therapeutic efficacy, (ii) the potential to impede or delay the onset of resistance, (iii) dose reduction, whilst maintaining potency, and avoiding adverse effects, and (iv) the potential for selective synergism or toxic antagonism, were realised much sooner for diseases like cancer, AIDS and tuberculosis [6–8]. Thus, it follows that the methods used to analyse drug interactions have been evaluated extensively and owe their origins to these disciplines. However, the lack of a standardised definition for synergy has resulted in confusion and conflict in the field, leading to many unsubstantiated claims of synergistic potential [8, 9].

We recently reported the findings of a repositioning screen on selected leads from FDA-approved compound libraries, which identified the anti-amoebic drug emetine dihydrochloride to have potent antimalarial efficacy [10]. SYBR Green fluorescence flow-cytometric analysis of K1 *P*. *falciparum* isolates showed ED_50_ values of 47 nM (44.9–49.2) for emetine dihydrochloride [10]. The natural product anti-protozoal drug (derived from the root of *Carapichea ipecacuanha*), was widely used in the treatment of hepatic and intestinal amoebiasis until it was replaced in the 1980s by the safer drug, metronidazole. The restriction of its medicinal use was due to side effects of nausea and vomiting following oral administration and dose-dependent cardiotoxicity at the higher doses required for use as a tissue amoebicide [11, 12]. The comparative *in vitro* ED_50_ data for *Entamoeba* species is reported at 26–32 uM [13] as opposed to 47 nM for the multidrug-resistant K1 [10] and 1 nM for the drug-sensitive 3D7 *P*. *falciparum* parasite lines [10, 13, 14]. Clearly, the dose-dependent toxicity profile for the repositioned use of emetine in malaria is likely to be varied and therefore merits further investigation. Defining combinatorial partner drugs with synergistic potential could improve the dose-dependent toxicity profile of this potent lead by widening therapeutic indices.

Indeed, synergy can occur as a result of the drugs interacting physically or most often as a consequence of each drug interacting at a molecular level with (same or different) components of the target [1, 15]. In attempting to identify the desirable occurrence of synergy, researchers have realised that the definition of additivity is most important. Synergism and antagonism are referred to as a ‘more than expected’ or ‘less than expected’ additive effect, respectively [9]. The majority of interaction methods are based on two definitions of additivity, namely, Bliss independence and Loewe additivity. Bliss independence (fractional product method) assumes that each drug acts independently. Therefore if two drugs A and B, each inhibit at 60%, additivity = 1- [(1-A)(1-B)], in this case 1-[(1–0.6)(1–0.6)] = 0.84. Thus to be additive, A + B would be expected to achieve 84% inhibition and deviation above or below this would be considered as synergistic or antagonistic respectively. Loewe additivity on the other hand, dictates that a drug cannot interact with itself. Therefore if in fact A and B were the same drug, the interaction can only be additive so 0.5A and 0.5B should be expected to achieve the same effect as drug 1A or 1B [16, 17]. Drug combinations are therefore additive if inhibition is constant along a line of equal effective dose. Hence the approach uses a line of constant inhibition (isobole) to define additivity. Deviations above the line (convex) are antagonistic and deviations below the line (concave) synergistic. Most commonly, the malaria community have classified drug interactions using the Fractional Inhibitory Concentration (FIC) data based on two outputs; the isobologram and sum of the FIC (∑FIC) value where <1 is synergism, equal to 1 is additivity, >1 is antagonism. However, the lack of standardised definitions between different research groups, particularly with regards to ∑FIC cut-offs for classifying an interaction as synergistic, additive or antagonist has meant that inconsistent, often biased, findings have appeared throughout the literature [5, 7, 18, 19]. The problem is further potentiated by the variation in treatment regimens used, such as fixed-ratio and checkerboard dosing, which means that irrelevant comparisons are often drawn between different ratios and dose levels (2, 9, 15–17). This is not unique to malaria drug combination studies and has in fact plagued the field of drug combinatory analysis. The lack of consensus prompted efforts by Chou and Talalay to resolve the issue [6, 9]. Arguing that synergy is a physiochemical rather than statistical issue, the Chou-Talalay method employs the mass-action law principle to derive a median-effect equation, (a unified theory derived from four existing physiochemical equations namely; Henderson-Hasselbalch (pH ionization), Hill (higher order ligand binding saturation), Michaelis-Menten (enzyme kinetics), and Scatchard (receptor binding)). The resulting combination index (CI) enables the quantitative definition of additivity (CI = 1), with <1 or >1 assigned as synergism and antagonism respectively. To eliminate subjectivity and permit automated data analysis, the complex algorithms for median-effect analysis were incorporated into a series of software programmes (CalcuSyn, CompuSyn), and are now widely used in biomedical science [6, 8, 17]. The entire shape of the growth inhibition curve is taken into account and the effects of the drug combination are quantified to see if they have greater effects together than expected from a simple summation of their individual effects [6]. Applying an automated, non-subjective method to malaria drug interaction studies would help standardise interaction classifications and permit more reliable comparisons between research groups.

In order to evaluate the method for antimalarial combinations, our preliminary work defined interactivity in a drug combination (atovaquone-proguanil) known to be synergistic against the malarial parasite [20, 21]. Dose-response curves and interaction assays were carried out in *in-vitro* cultures of the multidrug resistant K1 *Plasmodium falciparum* parasite line. The methodology was subsequently applied to investigate combinatorial regimes for the potent antimalarial candidate emetine dihydrochloride hydrate with a view to improving its toxicity profile. The work also introduces and validates use of the CalcuSyn software based on the Chou-Talalay method for the definition of antimalarial drug interactivity.

## Materials and methods

### Culture of *Plasmodium falciparum*

Erythrocytic stage, strain K1, *P*. *falciparum* parasites (gifted by Prof John Hyde, University of Manchester, UK, original source: Thai-K1 clone) were cultured in RPMI 1640 1x (+) L-Glutamine (+) 25 mM Hepes (Gibco, Life Technologies, UK) supplemented with 5% (w/v) lipid rich bovine serum albumin (Albumax II, Gibco, Life Technologies, New Zealand), 5 ng/ml hypoxanthine (Sigma, UK), 0.2% (w/v) glucose (Dextrose Anhydrous, Fisher Scientific, UK) and 50ng/ml gentamycin (Sigma, UK) at 37°C, under a 5% CO_2_ 5% O_2_ and 90% N_2_ gas mixture (BOC Limited, UK) in accordance with Read and Hyde, 1993 [22]. The parasites were routinely maintained in O+ human blood (NHS Blood Bank, Manchester, UK). All additives were sterile filtered at 0.22μm porosity and the complete medium was stored for no more than 2 weeks at 2–8°C. Continuous cultures were maintained at 5% haematocrit. Sorbitol was used to maintain synchronicity of the culture. Briefly, pelleted parasites (3,400 rpm for 5mins) were re-suspended 1:10 in sterile filtered 5% sorbitol ((w/v) in distilled water) and incubated at room temperature for 5 mins. Prior to continuation of the culture at 5% haematocrit, synchronised parasites were washed 3 x with complete media (3,400 rpm for 5mins). For experimental set up in a 96-well-plate format final well volumes were 200 μl at 2.5% haematocrit unless stated otherwise.

### Drug preparation

All compounds were obtained from Sigma Aldrich, UK. Primary stock solutions were prepared in accordance with manufacturer instructions. In brief, atovaquone (MW = 366.84) was dissolved at 5 mg/ml (13.63 nM) in DMSO. For dihydroartemisinin (MW = 284.35) 1.4 mg of the powder stock was dissolved in 1 ml of DMSO to obtain a primary stock concentration of 5 mM. Proguanil was dissolved at 1 mg/ml in acetonitrile: water (60/40). All primary stock solutions were passed through a 0.22 μm porosity filter, aliquoted and stored at -20°C until further use. Chloroquine (MW = 515.86) was prepared freshly on the day of use. An initial stock solution was prepared at 5mM (10.32 mg/ 4 ml) and sterile filtered. For experimental set up the primary stock solutions were further diluted with complete medium to give final test concentrations.

### Drug dosing for ED_50_ determination

Following preliminary screens (data not shown), refined dose ranges were selected for chloroquine (100–350 nM), atovaquone (0.64–40.89 nM), and proguanil (3.77–241.22 μM) to permit accurate ED_50_ calculation against the *P*. *falciparum* strain K1. Trophozoite stage parasites (0.5–1%) were treated for 48 hours and parasitaemia was determined using the SYBR Green flow cytometer method.

### SYBR Green staining of erythrocytic stage *P*. *falciparum* for flow cytometry

Following the drug treatment period in a 96 well plate format, an aliquot of each sample (100 μl) was transferred to a micro-centrifuge tube and washed once with 1 ml 1xPBS (centrifugation: 90 seconds at 14, 000 rpm). Each pellet was re-suspended in 1 ml 5 x SYBR Green 1 solution (in 1xPBS) and incubated in the dark at room temperature. After 20 minutes, the staining solution was removed (90 seconds centrifugation at 14,000 rpm) and the pelleted samples were re-suspended in 250 μl of the fixation solution (0.37% formaldehyde in 1xPBS, formaldehyde solution for molecular biology 36.5%, Sigma, UK) and incubated at 4°C for 10–15 minutes. Subsequently the samples were washed 3 x in 1xPBS and finally suspended in 1 ml of 1xPBS. Flow cytometric analysis was based on SYBR green fluorescence determined by the FITC channel (Blue laser, excitation laser line 488 nm EX_max_ 494 nm/Em_max_520 nM) and cell size (forward scatter, FSC-A) using the BD FACsVerse flow cytometer. Fifty thousand events were recorded and there were three replicas of each sample. Fluorescent events in drug treated samples were compared with infected and uninfected blood counterparts and gated accordingly to obtain the percentage parasitaemia.

### Derivation of dose-response curves and ED_50_ values

For each replicate, percentage parasitaemia of drug treated samples was calculated relative to the infected controls, which were adjusted to equate to 100% growth. GraphPad Prism 5.0 was used to normalise the data so that the largest value in the data set corresponded to 100% and the smallest value corresponded 0%. Log-transformed drug concentrations were then plotted against the dose-response and the ED_50_ values were determined using nonlinear regression log (inhibitor) vs. Normalised response-Variable slope (GraphPad Prism 5.0).

### Comparison of CalcuSyn-based and FIC analysis of the dihydroartemisinin-emetine combination

After determination of the ED_50_, a dose range of 0.125–8 x the ED_50_ was established by a two-fold serial dilution. For dihydroartemisinin the doses ranged from 6.3nM to 40nM. For emetine the doses ranged was from 63 nM to 400 nM. Co-administration of the compounds occurred at each level, for example ED_50_ of dihydoartemisinin was combined with the ED_50_ of emetine and 2 x ED_50_ DHA was combined with 2 x ED_50_ Eme and so forth. Parasites were treated at trophozoite stage and incubated for 48h in a 96 well plate format. The SYBR Green flow cytometer was used to determine drug susceptibility. Triplicate data was converted to an averaged percentage and analysed for the median-effect using CalcuSyn software (Biosoft).

### CalcuSyn interaction assay for malaria

Drug interactions were determined using the isobologram and combination index method derived from the median-effect principle of Chou and Talalay (CalcuSyn software, Biosoft: Chou, 2010). The mathematic model, established to assess drug-drug interactions, employs the median effect principle formula f_a_/f_u_ = [D/D_m_]^m^ where fa is the fraction of cells affected, f_u_ = 1-f_a_, the fraction unaffected, D equals the concentration of the drug, D_m_ the drug dose required for 50% inhibition, and m the slope of the median effect curve. The isobologram which provides a graphical representation of the pharmacological interaction is generated by a straight line connecting the F_a_ points against the fixed ratio combinations of Drug 1 and Drug 2 on the x and y axes.

The combination index (CI) is a quantitative representation of the pharmacological interactivity which takes into account both the potency (Dm, ED_50_) and the shape of the dose response curve. CI for the classic isobologram (CI = 1) is derived from the formula CI = (D)_1_/(D_x_)_1_ + (D)_2_(D_x_)_2_ + (D)_1_(D)_2_/(Dx)_1_(D_x_)_2_ where (D_X_)_1_ and (D_X_)_2_ are the concentrations for Drug 1 and Drug 2 resulting in X% inhibition when acting singly and (D)_1_ and (D)_2_ the concentrations of the respective drugs when acting in combination to achieve the same (iso-effective) percentage inhibition [23]. The CI was generated by the CalcuSyn software over a range of f_a_ levels at different growth inhibition percentages. A CI was interpreted in accordance with Table 1.

**Table 1:** Combinatory index values, recommended symbols and descriptions for classifying synergism or antagonism using the Chou-Talalay method.

Range of CI	Symbol	Description
<0.1	+++++	Very strong synergism
0.1–0.3	++++	Strong synergism
0.3–0.7	+++	Synergism
0.7–0.85	++	Moderate synergism
0.85–0.90	+	Slight synergism
0.90–1.10	±	Nearly additive
1.10–1.20	–	Slight antagonism
1.20–1.45	––	Moderate antagonism
1.45–3.3	–––	Antagonism
3.3–10	––––	Strong antagonism
>10	–––––	Very strong antagonism

The more broadly defined criteria suggest that the combination index, CI < 1, = 1, and > 1 indicate synergism, additive effect and antagonism, respectively (Source: CalcuSyn manual, Biosoft, 2006).

### SumFIC calculation and manual isobologram preparation

Combinatory data for the DHA-Eme interactions was analysed using the fraction inhibitory concentration method as described previously [10]. Briefly, the FIC values were calculated based on ED_50_, ED_75_ and ED_90_ values of the drugs alone and in combination at a constant-ratio of 1:1 (FIC = Fraction of drug concentration required to produce IC_50_ when used in combination /Fraction of drug concentration required to produce IC_50_ when used alone). The Isobologram was generated by plotting the FIC values for one drug against the other. SumFIC (∑FIC) data were determined by adding the FIC for each drug together at each level of inhibition (∑FIC = (IC_50_ of A in mixture/ IC_50_ of A alone) + (IC_50_ of B in mixture/ IC_50_ of B alone)). ∑FIC criteria are highly varied in the literature. Applying the most stringent criteria, a ∑FIC of 1 was interpreted as additive, >1 as antagonism and <1 as synergy [24].

### Validation of the CalcuSyn assay for malaria

In order to validate the CalcuSyn method as a useful tool for analysing drug interactions against the malaria parasite, the known synergistic drug combination, atovaquone and proguanil, was used. The initial experiment adopted the treatment regime outlined above and the doses selected were based on the respective ED_50_ values obtained previously for atovaquone (0.24 nM-15.27 nM) and proguanil (1.98 μM-126.81 μM). Triplicate samples were analysed by SG-FCM after 48 h.

Due to the lack of a synergistic interaction predicted by the initial data the following optimization steps were trialled: firstly, the activity of the drug stocks was checked (data not shown). Secondly, the experiment was repeated with an increased atovaquone dose series to account for a slight shift in the ED_50_ (0.48 nM-30.53 nM). Thirdly, the treatment duration was adjusted from 48h to 72h and initiated against ring stage rather than trophozoite stage parasites. This experiment was simplified by selecting only three dose points (low, mid and high) for each compound: namely 2.18, 4.36 and 8.72 nM for atovaquone and 7.93, 15.85 and 31.70 μM for proguanil. The final experiment employed an 8-point dose series, incorporated the optimised parameters, (initiation at ring stage and 72h incubation) and reduced the atovaquone dose was (0.27 nM-17.01 nM) to account for the higher potency identified with the longer treatment period. Drug-susceptibility in all of the optimisation experiments was analysed using the SYBR Green-based Flow Cytometer Method (SG-FCM) method.

### Recovery of parasites treated with the atovaquone-proguanil combination

Since the dose response curves have failed to achieve > 90% inhibition, and different drugs and combinations plateau at different levels of inhibition, the atovaquone-proguanil combination was used to evaluate the accuracy of the SYBR Green FCM method at determining parasite viability after 72 hours of incubation. In brief, three dose points were selected for each compound that were previously shown to equate to ED_25,_ ED_50_ and ED_75_ levels of inhibition for a 72h treatment period (0.05, 0.2 and 0.8ng/ml for atovaquone and 2.4, 4.6 and 9.2 μg/ml for proguanil). Compounds were combined at all doses (ATQ + PG combinations) and administered singly as controls. Untreated and 3.75% acetonitrile: water (60:40) (proguanil solvent) controls were also included in the experiment. All treatments were set up in triplicate. The plate was incubated under conditions described previously and analysed at 72h using the SG-FCM method. Following the 72h treatment period, the drug/drug combinations were removed and a limiting serial dilution was performed for each sample (in triplicate). In brief, 50 μl samples of each treatment were transferred to microcentrifuge tubes and washed 3 x in 1 ml of complete media. The pellet was re-suspended in 100 μl of complete media so that the haematocrit was adjusted from 2.5% to 1.25%. Four 96 well plates were prepared by adding 200 μl of 1.25% haematocrit to each well. Seven 10-fold limiting serial dilutions were performed by adding 20 μl of each sample to row A, mixing thoroughly and transferring 20 μl to row B and so forth until row H where the excess 20 μl was discarded. On day seven 100 μl of spent media was removed from each well and replaced with fresh media and blood at 1% haematocrit, adjusting the final haematocrit to 1.75%. The plates were incubated for a further 10 days and analysed for growth using SG-FCM. In accordance with Sanz *et al*. (2011) the number of viable parasites was back-calculated from the day 20 data in a presence/absence manner using the formula X^n-1^ where n was the number of wells able to render growth (determined by SG-FCM) and was X the dilution factor [25]. The day 20 data was plotted for comparison with the 72 hour data for both the non-constant and constant ratio combinations.

### CalcuSyn analysis of emetine with chloroquine, dihydroartemisinin and atovaquone

Following validation of the Chou-Talalay method using the atovaquone-proguanil combination, the interaction between emetine and dihydroartemisinin was reanalysed using the optimised treatment regime. Subsequently the combinations between chloroquine-emetine and atovaquone-emetine were also analysed using the software. The doses used for each compound were based on previously determined ED_50_ values where the respective ED_50_ concentration served as the midpoint for a two-fold, constant-ratio dose series (Row E, Table 2).

**Table 2:** The dose series used for the combination of existing antimalarials (AM) with emetine (Eme).

Dose	Dose (nM)			
Level	Dihydroartemisinin	Chloroquine	Atovaquone	Emetine
**A**	0	0	0	0
**B**	0.625	28.75	0.136	6.25
**C**	1.25	57.5	0.272	12.5
**D**	2.5	115	0.544	25
**E**	5	230	1.088	50
**F**	10	460	2.175	100
**G**	20	920	4.35	200
**H**	40	1840	8.7	400
**AM:Eme ratio**	1:10	4.6:1	1:46	—

The constant-ratio for each combination between the existing antimalarial (AM) and Emetine dihydrochloride hydrate (Eme) is also shown.

## Results

### Determination of dose-response curves and ED_50_ values

**Fig 1:**
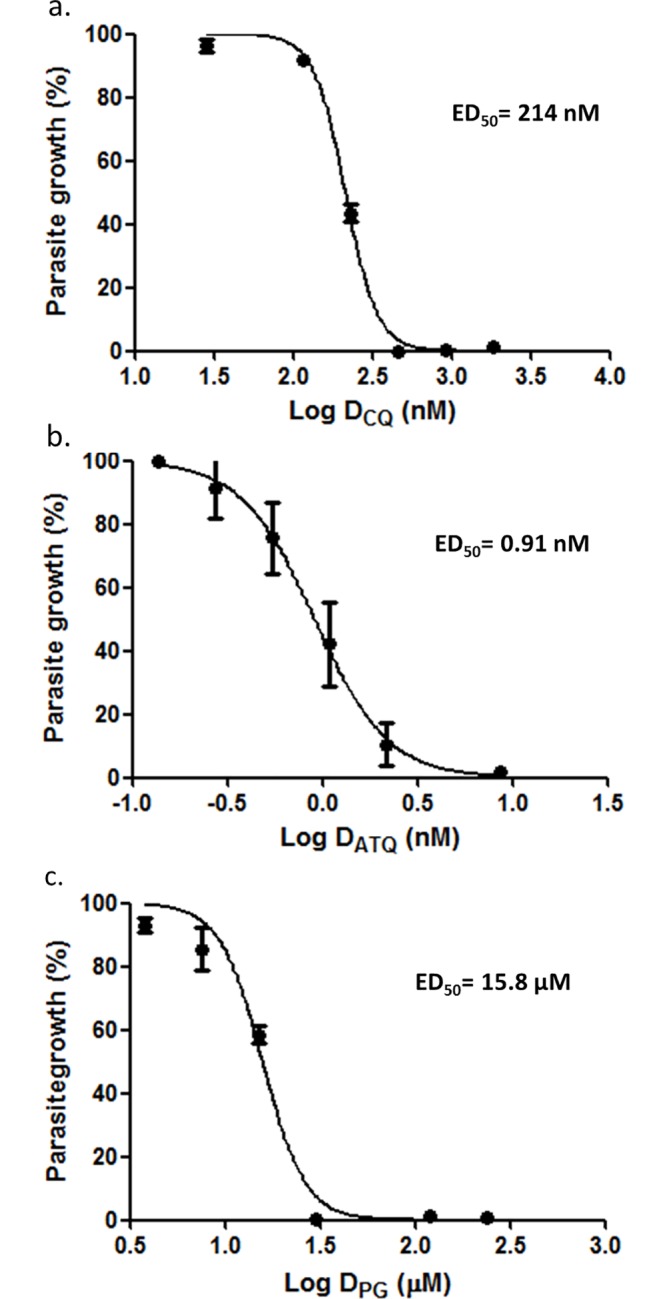
**Dose-response curves and ED**_**50**_ **values for a. chloroquine (CQ), b. atovaquone (ATQ) and c. proguanil (PG) against *P*. *falciparum*, strain K1.** Synchronised trophozoite stage parasites were treated with a two-fold dose series of the test compounds and analysed at 48 hours using the SYBR Green flow cytometer method. Standard error bars are based on the standard error of the mean (SEM) for triplicate data.

Initially dose-response curves were generated using the SYBR Green flow cytometer (SG-FC) method to permit accurate ED_50_ determination of all compounds used in the current study. Dose-response curves for dihydroartemisinin and emetine dihydrochloride hydrate have been presented previously with ED_50_ values of 2.6 nM (2.3–3.17) and 47 nM (44.9–49.2) respectively [10]. The ED_50_ values of chloroquine, atovaquone and proguanil presented here (Fig 1) were consistent with those reported elsewhere for the multidrug resistant, *P*. *falciparum*, strain K1 [24, 26].

### Comparison of CalcuSyn-based and FIC analysis of the dihydroartemisinin-emetine combination

**Fig 2:**
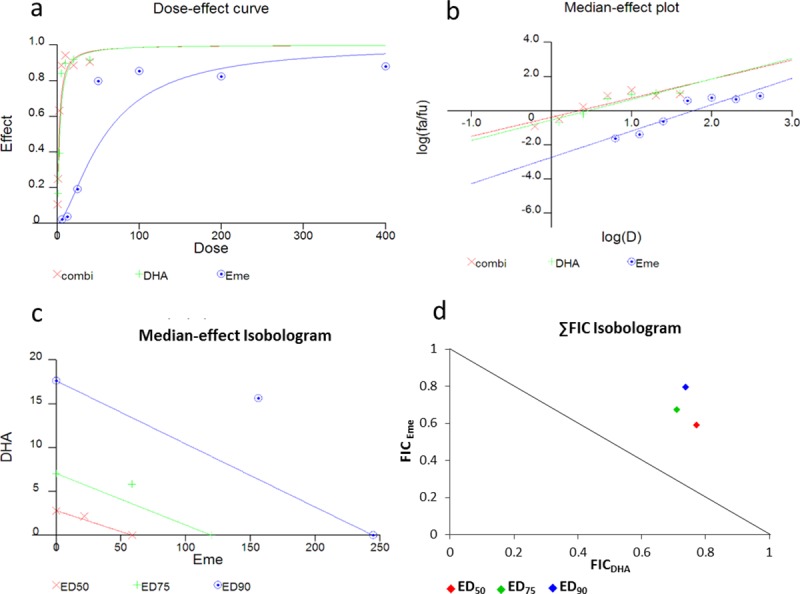
Comparison of dose-effect and FIC analysis for the dihydroartemisinin-emetine constant-ratio combination. Parasites were treated with a dose series of chloroquine, emetine and the combination. Dose ranges for individual and combined drugs were completed in triplicate. Dose-effect analysis was completed in accordance with Chou Talalay method using CalcuSyn. An isobologram was also prepared manually from the same, constant-ratio (ED_50_: ED_50_), dose-response data based on ∑FIC analysis. The dose-effect curve (a), the median-effect plot (b) and the isobologram (c) from the CalcuSyn output are shown alongside the FIC isobologram (d) For the ED_50_ ED_75_ and ED_90_ levels of inhibition.

The results show that similar interactivity classifications were obtained using a constant-ratio combination of dihydroartemisinin-emetine (1:10 dose ratio based on ED_50_: ED_50_ also considered 1:1 in some studies) when analysed using manual isobologram preparation and ∑FIC calculation Vs. automated CalcuSyn-based analysis. The interactivity determinants for the CalcuSyn analysis, the combinatory index (CI, equivalent to ∑FIC) and the isobologram plot, are based on both drug potency and shape of the dose-effect curve. In addition, the output includes a dose-effect curve and a median-effect plot to provide a figurative depiction of compound potency and conformity to the mass action law, respectively (Fig 2A and 2B). When the isobolograms and either ∑FIC or CI data were considered, both analyses indicated a predominantly antagonistic interaction between dihydroartemisinin and emetine across the inhibitory levels (Fig 2C and 2D). Specifically, ∑FIC = 1.36, 1.38 and 1.53, whilst CI = 1.15, 1.32 and 1.53 at the ED_50_, ED_75_ and ED_90_ levels, respectively. Interactivity classification applied to ∑FIC values are highly varied in the literature whilst CI criteria adhere to strict pre-defined classification ranges and infer slight antagonism at ED_50,_ moderate antagonism at ED_75_ and antagonism at ED_90_ (Table 1). Good correlations coefficients of the median-effect plot were reported for dihydroartemisinin (r = 0.91), emetine (r = 0.93), and the combination (r = 0.89), inferring good conformity to the mass-action law (Table 3).

**Table 3:** CalcuSyn output for the dihydroartemisin-emetine combination.

Drug	ED_50_	ED_75_	ED_90_	Dm	m	r
FIC_DHA_	0.772	0.710	0.736	N/A	N/A	N/A
FIC_DHA_	0.592	0.675	0.796	N/A	N/A	N/A
∑ FIC_DHA+Eme_	1.363	1.384	1.534	N/A	N/A	N/A
DHA	N/A	N/A	N/A	2.817	1.199	0.911
Eme	N/A	N/A	N/A	58.774	1.540	0.925
CI_DHA+Eme_	1.147	1.316	1.526	2.184	1.116	0.887

The combinatory index values (CI) potency at ED_50_ (Dm), shape of the curve (*m* = 1, > 1, and < 1 signify hyperbolic, sigmoidal, and flat sigmoidal respectively) and the linear correlation coefficient (r) presented in the table accompany the CalcuSyn output data presented in Fig 2 for the dihydroartemisinin-emetine combination against *P*. *falciparum*, strain K1 for 48 hours treatment at a constant ratio of 1:10.

### Optimisation of atovaquone-proguanil dosing and treatment period

**Fig 3:**
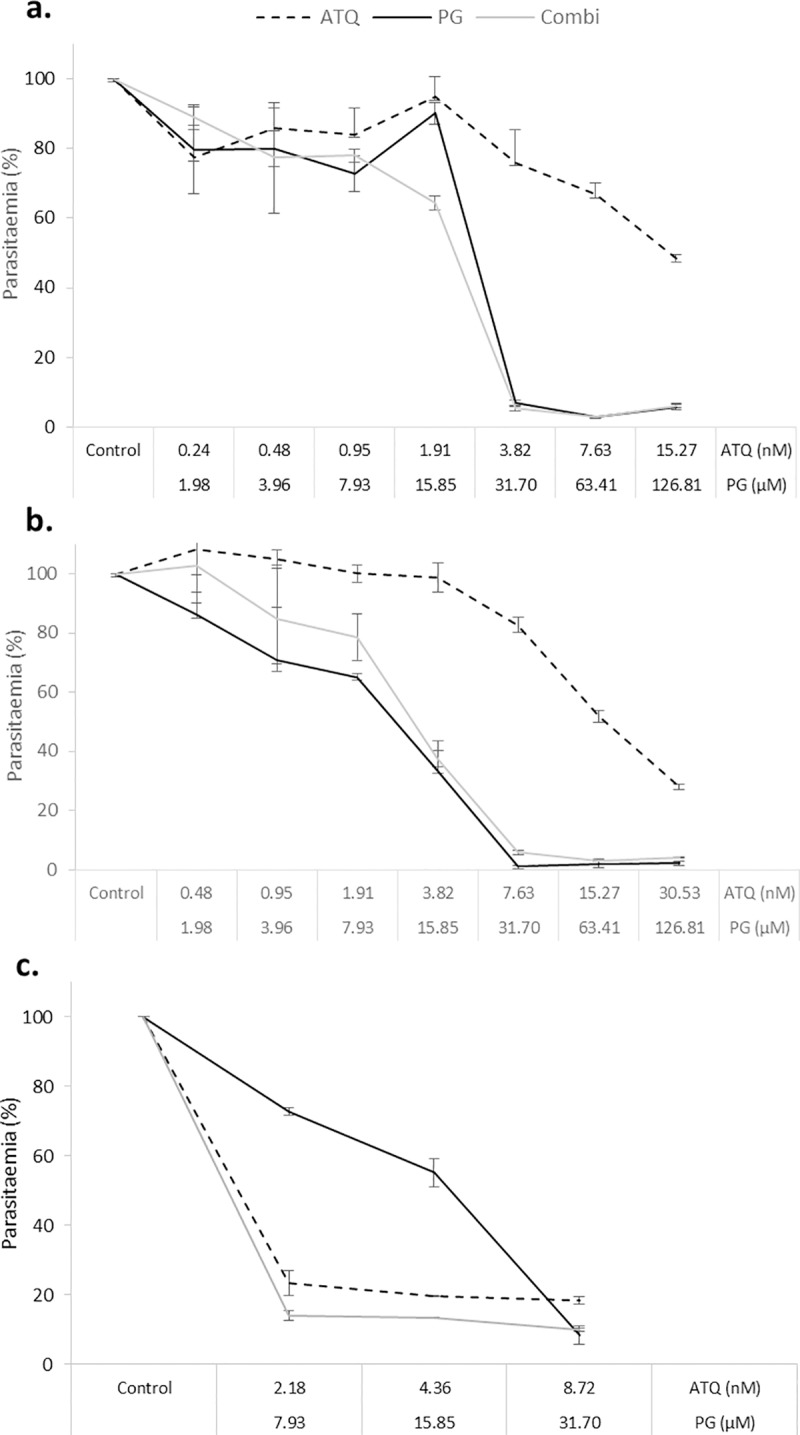
Optimisation of the atovaquone-proguanil treatment regime. (a) Trophozoite stage parasites (strain K1) were treated with a 2-fold dose series of atovaquone (ATQ), proguanil (PG) or a constant-ratio combination of both drugs (ATQ+PG) for 48 hours. The mid-point was equated to the previously determined ED_50_ value for each drug. (b) Was a replica of (a) but with an increased atovaquone dose series. (c) Ring stage parasites were treated for 72 hours with a 3-point constant-ratio dose series of atovaquone and proguanil either alone or in combination. SG-FCM method was used to analyse parasite growth. The parasitaemia of drug treated samples was determined relative to untreated controls (100% parasitaemia). Triplicate samples were used to derive error bars based on standard error of the mean (SEM).

**Fig 4:**
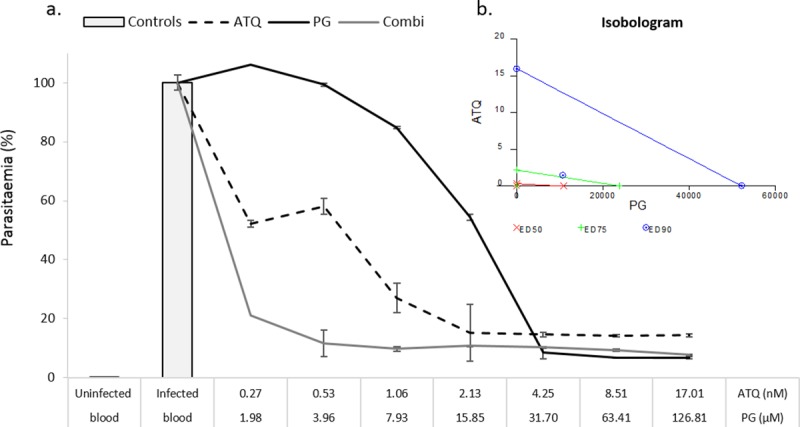
Constant-ratio combination of atovaquone and proguanil. The dose-response curves for atovaquone (ATQ), proguanil (PG) and the constant-ratio combination of the two drugs (a). Ring stage parasites were treated for 72 hours and analysed using the SG-FCM method. Controls consisted of uninfected blood, untreated controls and the highest dose of the acetonitrile: water (60:40) solvent for proguanil. Triplicate data were normalised according to the controls. Error bars reflect the standard error of the mean (SEM). The isobologram inset (b) shows the interactivity classifications for the constant-ratio combination of atovaquone and proguanil at the ED_50_, ED_75_ and ED_90_ effect levels.

The atovaquone-proguanil combination was selected as known antimalarial synergists to validate the CalcuSyn drug interactivity software for malaria. The compounds were combined under a constant-ratio in accordance with previously determined ED_50_ values (ED_50_:ED_50_). Initially, the combination failed to show a marked increase in growth suppression when compared with the drug-alone counterparts and the atovaquone dose-series did not achieve expected level of inhibition (Fig 3A). Despite a subsequent increase in dose-series, and up to 80% inhibition with atovaquone, a similar dose-response pattern emerged, and no evidence of synergy was apparent (Fig 3B). In both cases, inhibition for the combination was comparable to that of proguanil alone (Fig 3A and 3B). Finally, the extension of the treatment period from 48 hours to 72 hours did reveal a slight increase growth suppression in the combination, had the effect not been masked by the higher potency of atovaquone (Fig 3C). Accordingly, the reduction of the atovaquone dose in the subsequent experiment and increase to 72 hour incubation provided data whose analysis revealed the expected ‘very strong synergism’ interaction using CalcuSyn at the ED_50_ and ED_75_ levels of inhibition and ‘strong synergism’ at the ED_90_ level of inhibition (Fig 4 and Table 4).

**Table 4:** CalcuSyn output for the combination between atovaquone and proguanil.

Drug	CI Values at					
	ED_50_	ED_75_	ED_90_	Dm	m	r
ATQ	N/A	N/A	N/A	0.293	0.549	0.879
PG	N/A	N/A	N/A	10960	1.408	0.962
ATQ+PG (1:7455)	<0.001	0.004	0.295	2.2x10^-5^	0.198	0.825

The combinatory index values (CI) are shown for the ATQ + PG constant ratio combination (1:7455) and the ED_50_, ED_75_ and ED_90_ levels of inhibition. The Dm, m and r values are also shown for all sets of data. Dm indicates potency and depicts the median-effect dose at the ED_50_ level of inhibition. The m value refers to the kinetic order and shape of the curve m = 1, > 1, and < 1 indicates hyperbolic, sigmoidal, and negative sigmoidal shape, respectively. The r value is the linear correlation coefficient for the median-effect plot and indicates conformity to the mass action law.

### Further evaluation of SYBR Green-based *P*. *falciparum* viability using the atovaquone-proguanil combination

**Fig 5:**
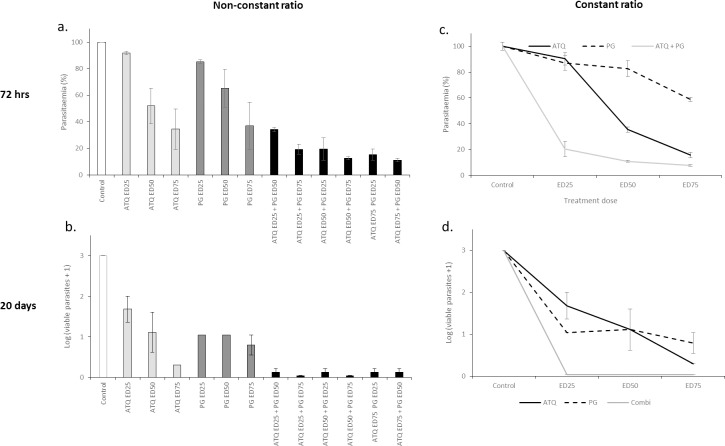
Non-constant and constant-ratio data for the atovaquone-proguanil combination. Ring stage parasites were treated for 72 hours with constant and non-constant ratio combinations of atovaquone and proguanil. Parasitaemia was determined using the SG-FCM method (a and c). After 72 hours of treatment, drug pressure was removed; parasites were subjected to 8 10-fold limiting serial dilutions and incubated for a further 17 days. Spent media was replaced with fresh media and erythrocytes on day 7. On day 20, the presence or absence of growth in each well was determined using SC-FCM and used to back calculate parasite viability after the 17 day recovery period (b and d). Triplicate data was used to construct error bars that represented the standard error of the mean (SEM).

In order to determine if parasite viability was misinterpreted by the SG-FCM method, parasite recovery and regrowth was monitored after exposure to constant ratio and non-constant ratio combinations of ATQ and PG. After 72 hrs of treatment the drugs were removed, parasites were subjected to a limiting serial dilution and allowed to recover for a further 17 days. The data showed that the combinations were more lethal than the drug alone treatments (Fig 5A–5D). After 72 hours of treatment the SG-FCM assay detected some remaining live parasites in all single and combined doses (Fig 5B and 5C), however, after 20 days, parasite growth was detected in the drug alone treatments but not in any of the constant ratio combinations (Fig 5D) There appears to be growth in some of the non-constant ratio dose treatments after 20 days. However in all cases growth was detected in only 1 out of 3 triplicates and the parasitaemia was only marginally above the background threshold of 0.2% parasitaemia (0.3, 0.2, 0.33 and 0.23 for ED_25_ ATQ + ED_50_ PG, ED_50_ ATQ + ED_25_ PG, EDC_75_ ATQ + ED_25_ PG and ED_75_ ATQ + ED_50_ PG respectively). The presence/ absence scoring system (viable parasites = X^n-1^) therefore skewed the data in these cases. Indeed control and drug alone treatments achieved parasitaemias of up to ~ 20% at the same dilution. A dose-response pattern could be observed in the drug alone treatments after 20 days of incubation confirming the reliability of the data. Although there appears to be the same level of regrowth at the ED_25_ and ED_50_ levels of inhibition for proguanil (growth present in the first two limiting serial dilutions for all concentrations) the parasitaemia showed a dose-response pattern. First dilution parasitaemias were 3.4% (SE ± 0.15) and 2.2% (SE ± 0.25) and second dilution parasitaemias were 0.83% (SE ± 0.06) and 0.60% (SE ± 0.03) for ED_25_ and ED_50_ doses respectively (control first dilution: 4.4% (SE ± 0.7) and second dilution: 3.7% (SE ± 0.16)). Although after 72 hours the highest dose of proguanil achieved nearly the same level of inhibition as the combinations, singly treated parasites were able to recover (after 17 days) whilst those subjected to the combinations were not.

### Defining the interactivity of emetine in combination with existing antimalarials

**Fig 6:**
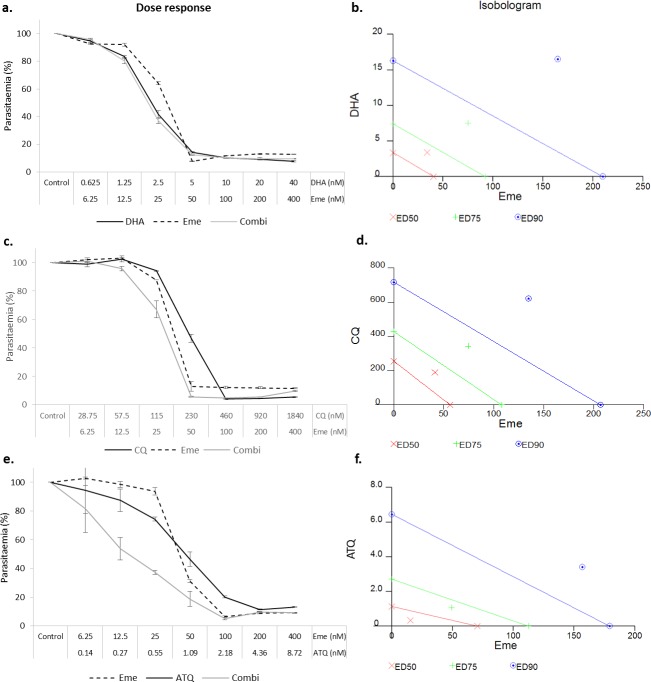
**The constant ratio combinations of dihydroartemisinin-emetine (a,b), chloroquine-emetine (c,d) and atovaquone-emetine (e,f) using CalcuSyn**. Parasite viability was analysed after 72 hours using the SG-FCM method. A basic dose-response curve is shown as well as the isobolograms to indicate the nature of the interaction for each constant-ratio (based on a 2-fold dose series where respective drug ED_50_: ED_50_ doses serveed as the midpoint) combination at the ED_50_ ED_75_ and ED_90_ effect levels (Marker below the line = synergistic, on the line = additive and above the line antagonistic). The degree of the classification is indicated by the distance of the marker from the respective (colour coded) line.

Three current antimalarial drugs, dihydroartemisinin (DHA), chloroquine (CQ) and atovaquone (ATQ) were investigated for their interaction with emetine dihydrochloride hydrate (Eme) using the optimised 72 hour treatment regimen. The constant-ratio for each combination was selected based on the respective predetermined ED_50_ values for each compound. The data for all of the combinations showed good conformity to the mass action law (r = 0.85–0.97). After 72 hours of incubation, the DHA-Eme interaction was classified as antagonistic (1:10 ratio) at all inhibitory levels analysed (Fig 6A and 6B, Table 5). The interaction between chloroquine and emetine (4.6:1 ratio) at ED_50_, ED_75_ and ED_90_ levels inhibition was classified as antagonism, antagonism and mild antagonism respectively (Fig 6C and 6D, Table 5). For the atovaquone-emetine (ratio 1:46) combination the ED_50_ level of inhibition was classified as synergism, the ED_75_ moderate synergism and ED_90_ moderate antagonism (Fig 6E and 6F, Table 5).

**Table 5:** Combinatory classification for emetine with existing antimalarials using the CalcuSyn analysis method.

Drug	CI Values at			
	ED_50_	ED_75_	ED_90_	r
DHA+Eme (1:10)	1.803	1.805	1.808	0.899
CQ+Eme (4.6:1)	1.469	1.489	1.517	0.877
ATQ+Eme (1:46)	0.516	0.834	1.403	0.912

The combinatory index values are shown for the combination at the ED_50_, ED_75_ and ED_90_ levels of inhibition. The r value for each combination is also reported to indicate the correlation coefficient of the data to the mass action law.

## Discussion

### Use of the Chou-Talalay method for *in vitro* drug interactivity analysis against malaria

Antimalarial interactivity analysis requires standardisation to overcome the contradictory classifications reported in the literature and the commonality of *in vitro-in vivo* disconnect. The ease-of-use of a constant-ratio treatment regime and automated CalcuSyn analysis, could serve as a standardised, objective starting point to analyse potential antimalarial combinations. Indeed, other areas of parasitology have adopted CalcuSyn as an alternative to manual isobologram preparation for both fixed-ratio and checkerboard data [27–30]. For malaria, the current study validated the use of CalcuSyn *via* comparison with Sum FIC data for the DHA-Eme interaction and identification of a synergistic interaction for the known antimalarial synergists, atovaquone and proguanil consistent with data reported elsewhere and in the field [5, 7, 20]. Importantly, the work highlighted that many factors, such as treatment time-course, viability assay and drug mode-of-action can confound interactivity classifications. Indeed, initially there was no evidence of an interaction between atovaquone and proguanil after 48 hours of treatment using the SYBR-Green-based flow cytometer assay. Solubility issues of atovaquone, similarly reported by Canfield and colleagues (1995)[5], were first considered. However, further work revealed that a 72 hour time-course was required for the identification of synergy. This was not surprising, as atovaquone is known to be one of the slowest acting antimalarials available [25], but contradicts the findings of Fivelman *et al*., (2004)[7], who showed the atovaquone-proguanil combination to be synergistic within a 48 hour treatment period using the hypoxanthine incorporation method. The problem may therefore be related to the impact of drug-kill profile on the viability determinant. Wein *et al*., (2010)[31] suggest that while the hypoxanthine incorporation method can be used at 48 hrs, the SYBR green-based methods require a 72 hour exposure period to provide accurate data, as the read-out can be differentially affected by drug mode-of-action, particularly cytocidal or cytostatic activities of drugs [31, 32].This notion is supported by the observations that no drug dose used in the study achieved 100% inhibition and different drugs plateau at different levels of inhibition, with the combination probably mirroring the most DNA degrading of the two compounds.

For this reason the ATQ PG combination was used to further understand the relationship between SYBR Green viability and cytocidal versus cytostatic effects of drugs at higher levels of inhibition. Following 72 hours of exposure with ATQ and PG alone and combination treatments an extended regrowth/recovery assay was conducted, in the absence of drug pressure. The assumption was that if the failure to exceed 90% inhibition was indeed due to background effects recovery would be consistent across all conditions with similar levels of inhibition at 72 hours. Interestingly, despite showing some remaining live parasites after 72 hours of treatment, comparable to that of some single drug treatments, parasites treated with any of the constant or non-constant ratio combinations did not recover to the same extent as their drug alone counterparts. The regrowth/recovery assay therefore revealed that the SYBR Green based flow cytometer assay misinterpreted parasite viability. At the highest levels of inhibition such errors are likely to influence the interactivity definition at arguably the most important levels of inhibition in terms of parasite killing[8]. It is unlikely that such problems are unique to the SYBR green assay, particularly as most drug-susceptibility assays, based on metabolic activity are suggested to offer poor surrogates for parasite viability and have also been reported to be differentially affected by the mechanistic actions of drugs [25, 31]. However, since most antimalarial combinatory analyses are conducted at the ED_50_ level of inhibition it is likely that such issues have been commonly overlooked by other screens. With such variability in experimental set up between screens, it is perhaps predictable that interactivity classifications reported in the literature are often inconsistent and translate poorly in the *in vivo* setting. Drug killing-profile data based on limiting serial dilutions, has been shown to generate *in vitro* pharmacokinetic (PK) and pharmacodynamic (PD) data that correlates well with *in vivo* PK/PD data [25]. Better *in vitro* to *in vivo* translation may therefore be achieved for drug combination analysis if more detailed second-phase *in vitro* drug combinatory analyses determined the drug interactivity outcome after drug wash-out and regrowth/recovery period, when parasite viability is definitive [18, 25, 33, 34].

A separate issue in terms of the *in vitro* to *in vivo* disconnect, is that *in vivo* combination ratios are affected by pharmacokinetic and pharmacodynamic aspects of the individual compounds. This makes the selection of an appropriate ratio at the *in vitro* level difficult, particularly, as in most cases, different ratios present different interaction classifications [8]. The value of identifying desirable interactions *in vitro* also becomes redundant if such ratios cannot be achieved in the *in vivo* situation. The CalcuSyn constant ratio option therefore serves as a good starting point, offering a user-friendly, objective route to achieve preliminary drug interaction data by employing a single-ratio, across a dose series, based on pre-defined ED_50_ values [6, 28, 30]. Second-phase combinatory analysis should therefore also include the evaluation of a wider range of pharmacokinetically appropriate drug ratios.

### Defining the interactivity of emetine in combination with existing antimalarials

The preliminary data predict that the DHA-Eme and CQ-Eme combinations would not be efficacious *in vivo*. Interestingly like emetine, chloroquine has also been used previously to treat amoebiasis [13]. In this protozoan parasite, the drugs were found to act on different parasite targets. Chloroquine was shown to inhibit DNA synthesis in the vegetative forms of the parasite, while emetine killed the trophozoite stage by inhibiting protein synthesis [13]. Indeed, emetine has been commonly reported as a protein synthesis inhibitor in other organisms including bacteria [11, 35]. Recent work by Wong and colleagues (2014) has solved the structure of the 80s *P*. *falciparum* cytoplasmic ribosome, with emetine bound to the E-site on the 40s subunit, confirming that such a target extends to the malaria parasite [36].

Interestingly, the combination between atovaquone and emetine was shown to be synergistic at ED_50_ and ED_75_ levels on inhibition but antagonistic at ED_90_ level. In other studies sporadic cases of antagonism in combinations that are otherwise synergistic have been reported [19]. However due to the previous findings, at high levels of inhibition where differences in inhibition are less pronounced the misinterpretation of parasite viability could be responsible for the contrary classifications presented. We have recently shown that similar to atovaquone, emetine has a slow killing profile against the malaria parasite (unpublished data). Interestingly, compounds with similar killing profiles have been shown to have similar parasite targets [25]. Atovaquone is known to act *via* the cytochrome bc1 complex in the malaria parasite mitochondria [37]. Coincidently, in the parasite, protein synthesis is known to occur at two other centres in addition to the cytosol: namely: the apicoplast and the mitochondria [36]. Both organelles are thought to have originated from separate endosymbiotic events of bacteria. Taken together, the relatively slow killing profile of emetine, mimicking that of atovaquone, the bacterial nature of the mitochondria and its involvement in protein synthesis infers that the preferential target of emetine may be the mitochondrial ribosome. The hypothesis would favour the synergistic activity reported here between the two drugs, possibly through activity on different targets in the mitochondria [15]. However such theories would require a more detailed inquiry and cannot be deduced from the data presented here alone. Nonetheless, preliminary evidence of a potentially pharmacokinetic-matched, synergetic combination presents an exciting opportunity for antimalarial development, providing dose-dependent toxicity can be overcome. Indeed, recent findings that atovaquone resistant parasites were not transmissible suggest the mitochondria may be a good drug target in terms of transmission intervention [38]. Further investigation will be needed to establish whether the combination will be efficacious *in vivo*.

Despite being identified as a recurring hit in high throughput screens [14, 39, 40], the value of emetine for malaria treatment has been largely overlooked due to concerns about its toxicity profile. The identification of a synergistic interaction between emetine and atovaquone further validates emetine dihydrochloride hydrate as a valuable antimalarial candidate and presents a potential route to minimise dose-related toxicity previously reported for the drug. Our further work will be focussed on evaluating other combinatorial options for the compound and attempting the synthesis of less toxic derivatives of the molecule in a bid to reduce undesirable, non-target effects. The recent and timely resolution of the cryo-EM structure of emetine in complex with its target on the E-site of the *P*. *falciparum* ribosomal small subunit, provides a useful tool to enable further modification of the molecule [36]. In the current backdrop of an impending void in the antimalarial drug market due to evolving artemisinin resistance, it is vital that powerful and affordable candidates like emetine are investigated further.
